# Antioxidant defense in the eyes of immunocompetent and immunosuppressed mice infected with *Acanthamoeba* spp.

**DOI:** 10.1186/s13071-020-3979-5

**Published:** 2020-03-07

**Authors:** Karolina Kot, Danuta Kosik-Bogacka, Patrycja Kupnicka, Natalia Łanocha-Arendarczyk

**Affiliations:** 1 Department of Biology and Medical Parasitology, Pomeranian Medical University in Szczecin, Powstancow Wielkopolskich 72, 70-111 Szczecin, Poland; 2 Independent of Pharmaceutical Botany, Department of Biology and Medical Parasitology, Pomeranian Medical University in Szczecin, Powstancow Wielkopolskich 72, 70-111 Szczecin, Poland; 3 Department of Biochemistry and Medical Chemistry, Pomeranian Medical University in Szczecin, Powstancow Wielkopolskich 72, 70-111 Szczecin, Poland

**Keywords:** *Acanthamoeba* spp., Eyes, Anti-oxidant enzymes, Immunological status

## Abstract

**Background:**

*Acanthamoeba* spp. are ubiquitous pathogens which cause granulomatous amoebic encephalitis and disseminated infection. Moreover, *Acanthamoeba* spp. infection of the cornea leads to *Acanthamoeba* keratitis. Our previous study showed that the infection of an eyeball may also take place *via* the migration of trophozoites through the optic nerve from the brain to the eyes. The aim of the study was to analyze the activity of enzymatic antioxidants and the concentration of non-enzymatic antioxidant in the eyes of immunocompetent and immunocompromised mice with disseminated acanthamoebiasis.

**Results:**

In the immunocompetent mice infected with *Acanthamoeba* spp. we noted a significant decrease in catalase activity at 8 and 16 days post-infection (dpi). Glutathione reductase activity was significantly lower at 16 dpi compared to the control group and glutathione concentration was statistically higher at 24 dpi than in the control group. In the immunosuppressed mice, a statistically significant increase in glutathione concentration in the eye samples was found at 16 dpi compared to those not infected with *Acanthamoeba* spp. In the immunosuppressed mice infected with *Acanthamoeba* spp., glutathione peroxidase activity was statistically lower at 8 dpi, and glutathione concentration was statistically significantly higher at 16 dpi compared to the control group.

**Conclusions:**

The inflammatory response in the eyes of hosts with experimental acanthamoebiasis led to changes in the activity of enzymatic antioxidants and the content of non-enzymatic antioxidant. Therefore, the dysregulation of antioxidants may play a role in the pathomechanism of *Acanthamoeba* eye infection.
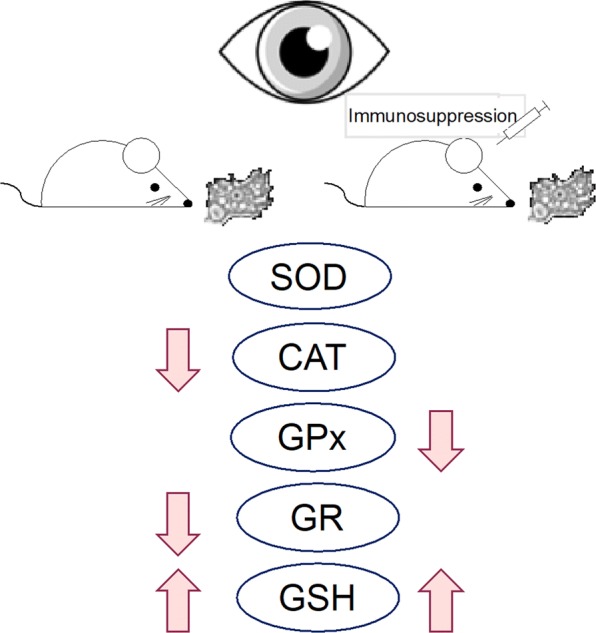

## Background

Reactive oxygen species (ROS) play role in the transmission of inter- and intracellular signals and in the destruction of pathogens by phagocytosis. Additionally, ROS regulate gene expression through the activation of transcription factors, which play an important role in the activation of the immune response [1, 2]. When the rate of ROS production exceeds the effectiveness of antioxidant defenses, e.g. during infection or inflammation, oxidative stress occurs [3, 4]. Thus, disrupted oxidative balance plays a significant role in the physiopathology of many parasitic diseases, such as pulmonary acanthamoebiasis [5].

*Acanthamoeba* spp. are ubiquitous pathogens which cause granulomatous amoebic encephalitis (GAE) and disseminated infection in the liver, kidneys and lungs in immunocompromised patients [6, 7]. Moreover, *Acanthamoeba* spp. invade the cornea of the eye, leading to *Acanthamoeba* keratitis (AK). Amoebae adhere to the surface of the cornea through acanthopods and adhesins present on the surface of the parasite cell, including mannose binding protein (MBP) and laminin binding protein [8, 9]. When amoebae come into contact with the cornea of the eye, adhesins interact with glycoproteins and glycolipids of the host corneal epithelium [10]. The amoebae penetrate the corneal epithelium, causing exfoliation through cell cytolysis, phagocytosis and apoptosis. The adhesion stage is followed by the secretion of enzymes and cytotoxic substances [11, 12]. *Acanthamoeba* trophozoites activate host matrix metalloproteinases (MMPs) leading to the degradation of basal membranes and produce serine and cysteine proteases [13] allowing the amoebae to invade the cornea of the eye, accompanied by inflammatory reactions, swelling and necrosis [8]. Proteases induce apoptosis in macrophage cells and degradation of keratocytes, ciliary body cells, retinal pigment epithelium, corneal epithelium and endothelial corneal cells [8]. The last stage of AK is inflammation of the cornea nerve fibres [10].

Our previous study has shown that the infection of an eye may also take place *via* the migration of trophozoites through the optic nerve from the brain to the eyes [14]. Moreover, we observed an altered expression of Toll-like receptors (TLR) 2 and 4 in the eyes of mice intranasally infected with *Acanthamoeba* spp. [14]. It was found that oxidative stress or dysregulation of antioxidants may upregulate the expression of TLR2 and TLR4 [15].

Laboratory findings indicate that oxidative stress and decreased effectiveness of the antioxidant defense system have been implicated in the pathogenesis of several eye conditions, such as corneal disease, cataract and macular degeneration [16, 17]. Patients with ocular diseases have lower levels of superoxide dismutase (SOD), catalase (CAT) and glutathione peroxidase (GPx) [18]. However, there is limited information on antioxidant enzyme activity during acanthamoebiasis. Łanocha-Arendarczyk et al. [5] showed a reduction in antioxidant capacity in hosts infected with *Acanthamoeba* spp., while Motavalli et al. [19] showed that under oxidative stress, the defense reactions of the parasite are in part mediated by increasing its antioxidant activity, which is important for the survival of the parasite. Hadaś & Mazur [20] showed a correlation between SOD activity and *Acanthamoeba castellanii* virulence.

In this study, a continuation of our previous study [14], we want to show that the dysregulation of antioxidants is a part of the pathomechanism during acanthamoebiasis. Therefore, we analyzed the activity of enzymatic antioxidants and the concentration of non-enzymatic antioxidant in the eyes of *Acanthamoeba-*infected immunocompetent and immunocompromised mice.

## Methods

### Mice

The experimental animal model has been described in our previous study [21]. In short, the *Acanthamoeba* spp. strain AM22 was isolated from a patient with acute myeloid leukemia (AML) and atypical pneumonia. Ninety-six mice were divided into four groups: uninfected immunocompetent control mice (C; *n* = 18); uninfected mice treated with an immunosuppressive drug (CS; *n* = 18); *Acanthamoeba* spp.-infected immunocompetent mice (A; *n* = 30); and *Acanthamoeba* spp.-infected mice treated with an immunosuppressive drug (AS; *n* = 30).

The immunosuppression, intranasal inoculation and pathogenic tests followed a previously described method [14]. The mice were sacrificed using sodium pentobarbital (Euthasol vet, FATRO, Raamsdonksveer, The Netherlands) injected intraperitoneally at 2 ml/kg body weight. The mice were weighed, and the eyes removed for analysis. The degree of *Acanthamoeba* spp. infection was determined after placing the eyes on NN agar and incubating at 41 °C [22]. Eye samples for biochemical analysis were stored at − 80 °C and those for histological analyses were stored in 4% buffered formalin solution (Avantor, Gliwice, Poland). As a continuation of our previous research [14], in this study we decided to analyze the activity/concentration of antioxidants in the eyes of the mice as they were too small to analyze ophthalmic tissues separately.

### Histological analyses

After dehydration in alcohols with increasing concentrations, samples of the eyes were embedded in paraffin and sliced (5 μm) using a microtome and stained with hematoxylin and eosin. Histological preparations were evaluated using a Zeiss light microscope at 400× and 1000× magnification.

### Biochemical analyses

#### Homogenization of samples

Frozen eye samples were first placed in a cooled metal homogenizer with liquid nitrogen, and then crushed with 4–5 strokes of a cooled metal mandrel. Frozen and powdered samples of approximately 1 mg of protein were then transferred into a cooled Eppendorf tube bucket containing 500 μl of suitable buffer at 4 °C (following the procedure of the Cayman Chemical, Ann Arbor, MI, USA of the kit for antioxidant enzymes and non-enzymatic antioxidants). After a short vortexing, blade homogenization was performed for 15 s. The extracted mixtures were centrifuged (3000× *g* for 10 min at 4 °C) and the supernatants were transferred to the freezer (− 80 °C) until analysis. Samples were then thawed at room temperature for analyses.

#### Superoxide dismutase (SOD) activity

The total activity (Cu-Zn and Mn) of superoxide dismutase was measured using a Superoxide Dismutase Assay Kit (Cayman Chemical, Ann Arbor, MI, USA) according to the manufacturerʼs procedure. The sample was first washed with 0.9% NaCl containing 0.16 mg/ml of heparin, then PBS was used to homogenize the sample. The method used is based on the increase in self-oxidation rate of a SOD-catalyzed reaction of 5,6,6a,11b-tetrahydro-3,9,10-trihydroxybenzo[c]fluorene in alkaline aqueous solution to a chromophore form with maximum absorption at 525 nm. To 40 μl of sample, we added 900 μl of 2-amino-2-methyl-1,3-propanediol buffer (pH 8.8), containing boric acid and diethylenetriaminopentaacetic acid and 30 μl of 1-methyl-2-vinylpyridine trifluoromethanesulfonate in HCl, and incubated for one minute at 37 °C. Then 30 μl of 5,6,6,6a,11b-tetrahydro-3,9,10trihydroxybenzo[c]fluorene was added. The reaction mixtures were transferred to the cuvettes. SOD activity was determined by measuring the absorbance at 450 nm using an Alpha 40 spectrophotometer (PerkinElmer, Waltham, USA). The activity was expressed in U/mg protein.

#### Catalase (CAT) activity

Catalase activity was measured using a Catalase Assay Kit (Cayman Chemical, Ann Arbor, MI, USA) according to the manufacturerʼs recommendations. The test is based on the assumption that the rate of hydrogen peroxide dismutation to water and molecular oxygen is proportional to the concentration of the catalase. Phosphate-buffered saline (PBS) (20 mM, pH 7.4) was used to homogenize the sample. The sample (30 μl) was incubated for one minute with 500 μl 10 mM H_2_O_2_ and the reaction was halted by adding 500 μl sodium azide. The reaction mixture (20 μl) was transferred to cuvettes and 2 ml of chromogen was added. CAT activity was determined by measuring the absorption at 540 nm using an Alpha 40 spectrophotometer (PerkinElmer). Catalase activity was expressed in U/mg protein.

#### Glutathione peroxidase (GPx) activity

Cellular glutathione peroxidase activity was measured using a commercial Glutathione Peroxidase Assay Kit (Cayman ChemicalAnn Arbor, MI, USA) according to the manufacturerʼs instructions. For homogenization of the sample, a buffer containing 1 mM mercaptoethanol, 5 mM EDTA and 5 mM Tris-HCl was used. In the test, an indirect measurement of enzyme activity was used. Oxidized glutathione (GSSG), which is the product of the GPx-catalyzed reaction, is converted to a reduced form (glutathione) by glutathione reductase at the expense of NADPH oxidation. To 350 μl of the sample we added 350 μl of 50 mM buffer containing 0.5 mM EDTA, 5 mM NADPH, 42 mM of glutathione and 10 units/ml of glutathione reductase. The reaction was initiated by the addition of 350 μl of 30 mM tertiary butyl hydroxide, then NADPH oxidation was measured by the decrease in absorbance at 340 nm using an Alpha 40 spectrophotometer (PerkinElmer). GPx activity was defined as the amount of sample required to oxidize 1 μM NADPH per minute, based on a molecular absorbance of 6.22 × 10^6^ for NADPH and expressed in U/mg protein.

#### Glutathione reductase (GR) activity

Glutathione reductase activity was determined using a Glutathione Reductase Assay Kit (Cayman Chemical, Ann Arbor, MI, USA) according to the manufacturerʼs instructions. PBS was used to homogenize the sample. The test is based on the oxidation of NADPH to NADP^+^ catalyzed by low concentrations of glutathione reductase. To 200 μl of the sample we added 400 μl of GSSG, followed by 400 μl of NADPH. GSSG reduction was determined indirectly by measuring NADPH consumption in the reduction of absorbance at 340 nm using an Alpha 40 spectrophotometer (PerkinElmer). GR activity was determined as the amount of sample required to oxidize 1 μM NADPH per minute based on a molecular absorbance of 6.22 × 10^6^ for NADPH and expressed in U/mg protein.

#### Glutathione (GSH) concentration

The concentration of reduced GSH was measured with a Glutathione Assay Kit (Cayman ChemicalAnn Arbor, MI, USA) according to the manufacturerʼs instructions. Glacial metaphosphoric acid was used for homogenization. Samples were stored at 4 °C and GSH concentration was determined over one hour based on the reactions of 4-chloro-1-methyl-7-trifluoromethylquinoline methyl sulphate and all mercaptans present in the sample, and on the elimination reaction in the alkaline medium which led to the formation of chromophoric cations showing maximum absorbance at 400 nm. To 40 μl of the sample we added 860 μl of potassium phosphate containing diethylene-triaminopentaacetic acid. Then 50 μl of chromogenic reagent and 50 μl of 30% NaOH were added. Samples were thoroughly mixed and incubated at 25 °C for 10 min in the dark. GSH concentration was determined by measuring absorbance at 400 nm using an Alpha 40 spectrophotometer (PerkinElmer) and calculated using standard curves.

### Determination of protein content

The activity of enzymes was calculated on the basis of protein content in the samples. Protein concentrations were measured with a MicroBCA Protein Assay Kit (Thermo Scientific, Peirce Biotechnology, USA) and plate reader (Asys UVM 340; Salzburg, Austria). The test kit is a two-component high-precision set of reagents for determining total protein concentration compared to a protein standard. This method combines the reduction of Cu^2+^ to Cu^+^ by protein in an alkaline environment and the sensitive colorimetric detection of copper cations at the first stage of oxidation using bicynchonic acid [23].

### Statistical analysis

Statistical analysis was performed using StatSoft Statistica 12.0 and Microsoft Excel 2016. For each of the examined parameters, the arithmetic mean (AM) and standard deviation (SD) were calculated. Differences between the examined parameters were assessed using the non-parametric Mann-Whitney U-test and Kruskal–Wallis test. Statistical differences were assumed to be significant at *P* < 0.05.

## Results

### Clinical symptoms of disseminated acanthamoebiasis in mice

In the immunocompetent and immunosuppressed mice infected with *Acanthamoeba* spp. (groups A and AS), changes in appearance were observed, such as emaciation, tousled fur and a hunched posture, as well as behavioural changes such as strong excitation, aggression and revolving movement. Five mice infected with *Acanthamoeba* spp. showed symptoms indicating imminent death, including reductions in temperature and body weight, impaired breathing and behavioural changes (including reduced movement). A humane endpoint was used for these animals.

### Histological changes in the eyes

**Fig. 1:**
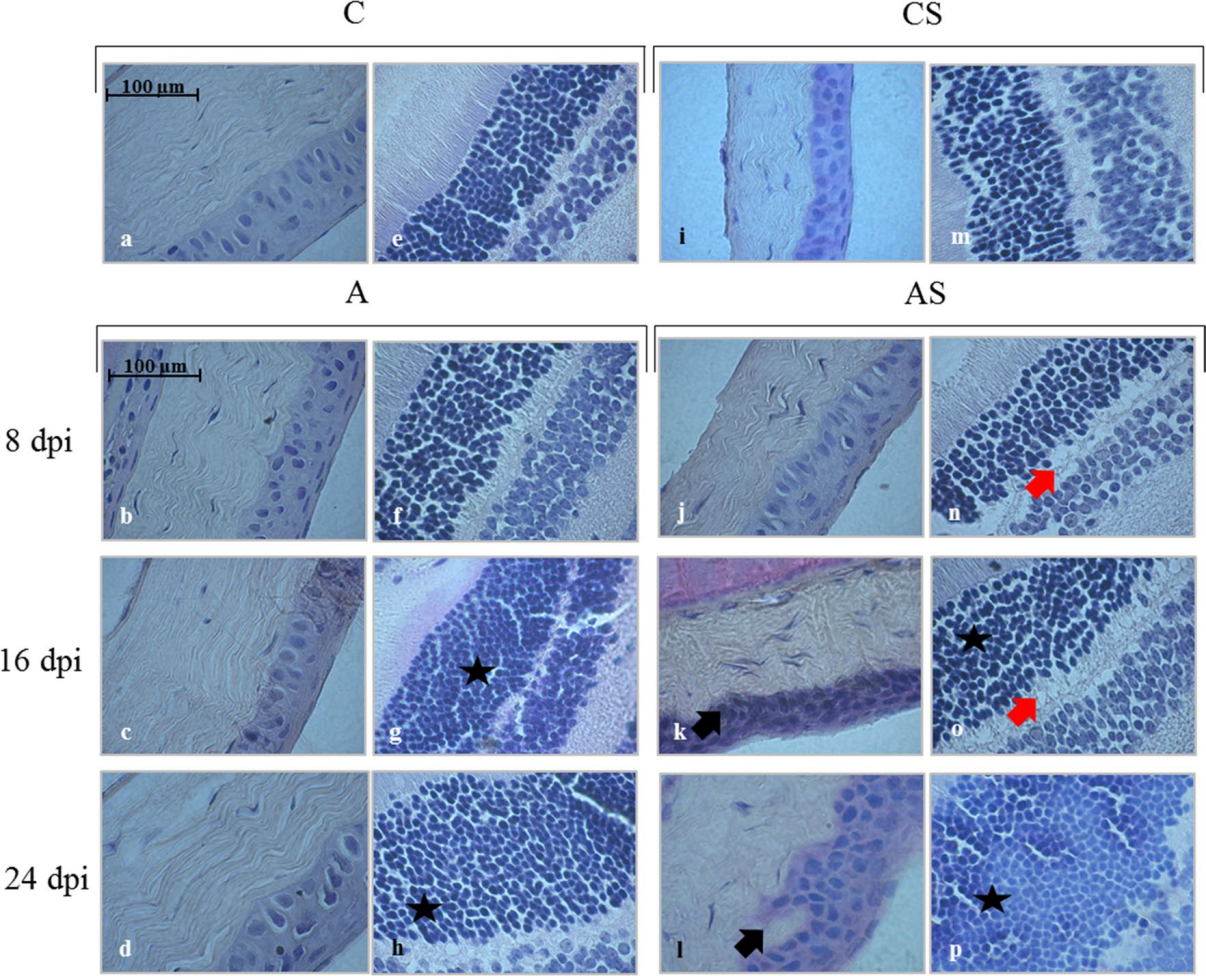
The cornea (**a**–**d**, **i**–**l**) and retina (**e**–**h**, **m**–**p**) in immunocompetent and immunosuppressed mice infected with *Acanthamoeba* spp. *Abbreviations*: A, immunocompetent mice infected with *Acanthamoeba* spp,; AS, immunosuppressed mice infected with *Acanthamoeba* spp,; C, uninfected immunocompetent mice; CS, uninfected immunosuppressed; dpi, days post-*Acanthamoeba* spp. infection. *Key*: black stars, increased thickness of outer nuclear layer; black arrows, invagination of the Bowmanʼs membrane into the substantia propria; red arrows, vacuolization inside the outer plexiform layer

Figure 1 presents the histological changes in the cornea and retina in the immunocompetent and immunosuppressed mice infected with *Acanthamoeba* spp. at 8, 16 and 24 dpi, and the uninfected mice. In the eye tissue of the immunocompetent infected mice at 16 and 24 dpi we observed an increased thickness of the outer nuclear layer (Fig. 1c, d; black stars). In the cornea immunosuppressed infected mice there was an invagination of the Bowmanʼs membrane into the substantia propria (Fig. 1k, l; black arrows). Moreover, in the chronic infection with *Acanthamoeba* spp. in the immunosuppressed mice there was an increased thickness of the outer nuclear layer in the retina (Fig. 1o, p; black stars), and an increase in vacuolization inside the outer plexiform layer that was absent in the other groups (Fig. 1n, o; red arrows).

In the eye tissues of the *Acanthamoeba* spp.-infected immunocompetent and immunosuppressed mice we did not find any developmental forms of *Acanthamoeba* spp.

### SOD activity

There were no statistically significant differences in SOD activity in the eyes of immunocompetent and immunosuppressed mice infected with *Acanthamoeba* spp. and animals of the respective control groups, despite the fact that there was a decrease in SOD activity in the eyes of immunocompetent mice at 24 dpi compared to control group (Table 1). A statistically significant between in SOD activity (*U*_(18)_ = 14, *Z* = − 1.90, *P* = 0.05) was only found in the eye samples at 24 dpi in both the infected immunocompetent and immunocompromised mice.

**Table 1:** Activity of peroxide dismutase, catalase, glutathione peroxidase, glutathione reductase and glutathione reductase in eye samples of immunocompetent and immunosuppressed infected and uninfected mice with *Acanthamoeba* spp

Parameter	C	CS	A	AS
SOD (U/mg protein)				
8 dpi	0.36 ± 0.14	0.37 ± 0.15	0.34 ± 0.16	0.36 ± 0.12
16 dpi			0.36 ± 0.14	0.33 ± 0.08
24 dpi			0.22 ± 0.09*	0.34 ± 0.06*
CAT (U/mg protein)				
8 dpi	7.96 ± 4.97*	7.46 ± 4.75	3.89 ± 2.26*	8.02 ± 3.74*
16 dpi			2.36 ± 1.49*	11.36 ± 6.32*
24 dpi			10.40 ± 3.43	6.68 ± 4.19
GPx (U/mg protein)				
8 dpi	4.62 ± 2.96	3.73 ± 1.87*	3.27 ± 1.75	1.31 ± 0.81*
16 dpi			2.38 ± 1.02	3.19 ± 1.66
24 dpi			2.36 ± 0.89	2.83 ± 1.07
GR (U/mg protein)				
8 dpi	3.77 ± 3.02*	2.46 ± 2.10	2.24 ± 1.69	0.98 ± 0.63
16 dpi			0.75 ± 0.88*	1.43 ± 1.06
24 dpi			2.15 ± 1.05	3.87 ± 3.72
GSH (mmol/mg protein)				
8 dpi	4.41 ± 1.29*	5.37 ± 2.12*	3.94 ± 0.89	4.23 ± 0.41
16 dpi			3.58 ± 0.18*	6.57 ± 1.67*
24 dpi			5.30 ± 1.02*	5.21 ± 0.98

*Statistically significant difference (*P* < 0.05)

*Abbreviations*: A, immunocompetent mice infected with *Acanthamoeba* spp.; AS, immunosuppressed mice infected with *Acanthamoeba* spp.; C, uninfected immunocompetent mice; CAT, catalase; CS, uninfected immunosuppressed; dpi, days post-*Acanthamoeba* spp. infection, GPx, glutathione peroxidase; GR, glutathione reductase; SOD, peroxide dismutase

### CAT activity

CAT activity in the eye samples of the immunocompetent mice had decreased at 8 and 16 day post*-Acanthamoeba* spp. infection compared to the uninfected immunocompetent mice, and then increased at 24 dpi, with a statistically significant reduction (*U*_(18)_ = 6, *Z = *− 2.06, *P* = 0.03) in CAT activity at 16 dpi (Table 1). In the immunosuppressed mice, CAT activity increased at 8 and 16 days post-*Acanthamoeba* spp. infection and then decreased at 24 dpi. No statistically significant differences were found in the immunosuppressed mice at 8, 16 or 24 dpi compared to the control group. A statistically significantly higher CAT activity was observed in the eye samples of the immunosuppressed mice than in the immunocompetent mice at 8 (*U*_(18)_ = 2, *Z* = − 2.09, *P* = 0.03) and 16 days post-*Acanthamoeba* spp. infection (*U*_(18)_ = 12, *Z* = − 1.94, *P* = 0.05).

### GPx activity

The highest GPx activity in the eye samples both in the immunocompetent and immunosuppressed mice eye samples was observed in the uninfected mice (Table 1). No statistical differences were found in the activity of GPx in the eye samples between immunocompetent uninfected and infected with *Acanthamoeba* spp. mice. GPx activity in the eye tissue of the immunosuppressed mice 8 days post-*Acanthamoeba* spp. infection was statistically lower (*U*_(18)_ = 9, *Z* = − 2.70, *P* = 0.004) than the immunosuppressed uninfected mice. There were no statistical differences in GPx activity between the immunocompetent and immunosuppressed mice at 8, 16 and 24 days post-*Acanthamoeba* spp. infection.

### GR activity

The highest GR activity in the eye samples of the immunocompetent and immunosuppressed mice was observed in control groups and 24 days post-*Acanthamoeba* spp. infection, respectively (Table 1). In the immunocompetent mice a significantly lower GR activity (*U*_(18)_ = 6, *Z* = − 2.15, *P* = 0.03) was found 16 days post-*Acanthamoeba* spp. infection compared to the control group, otherwise there were no other statistically significant differences.

### GSH concentration

The highest GSH concentrations in the eye samples of the immunocompetent and immunosuppressed mice were found at 16 and 24 days post-*Acanthamoeba* spp. infection (Table 1). In the immunocompetent mice at 24 dpi, the GSH concentration was statistically higher in comparison to the control group (*U*_(18)_ = 35, *Z* = 2.46, *P* = 0.01). In the immunosuppressed mice, a statistically significant increase (*U*_(18)_ = 43, *Z* = 2.07, *P* = 0.04) in glutathione concentration in the eye samples was found at 16 dpi compared to the uninfected mice.

### Analysis of the SOD/CAT and SOD/GPx ratios

**Fig. 2:**
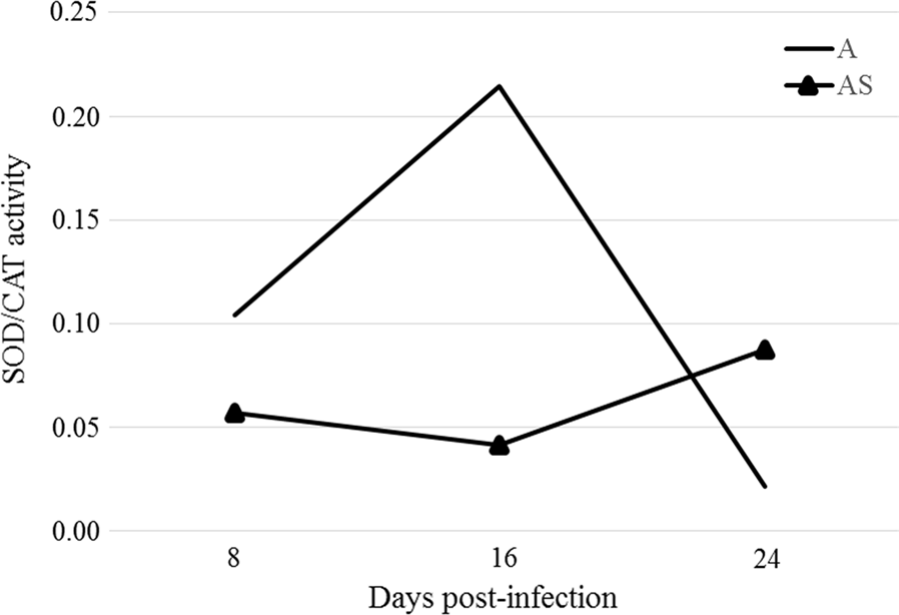
Ratio of superoxide dismutase activity to catalase in the eye samples of immunocompetent and immunosuppressed mice at 8, 16 and 24 days post-*Acanthamoeba* spp. infection. The graph shows arithmetic means. *Abbreviations*: A, immunocompetent mice; AS, immunosuppressed mice; CAT, catalase; dpi, days post-infection; SOD, superoxide dismutase activity

In the eye samples of the immunocompetent mice at 8 days post-*Acanthamoeba* spp. infection, a slight decrease in SOD/CAT ratio was observed, followed by a statistically significant increase at 16 dpi (*U*_(18)_ = 6, *Z* = 2.10, *P* = 0.03) compared to the control group, then a further decrease at 24 dpi (Fig. 2). In the immunosuppressed mice, a decrease in SOD/CAT ratio at the 8 and 16 days post-*Acanthamoeba* spp. infection and a slight increase at 24 dpi (Fig. 2) were observed, but the differences were not statistically significant. At 16 days post-*Acanthamoeba* spp. infection, the SOD/CAT ratio in the immunosuppressed mice was more than 5 times higher than in the immunocompetent mice (*U*_(18)_ = 3, *Z* = 2.08, *P* = 0.03).

**Fig. 3:**
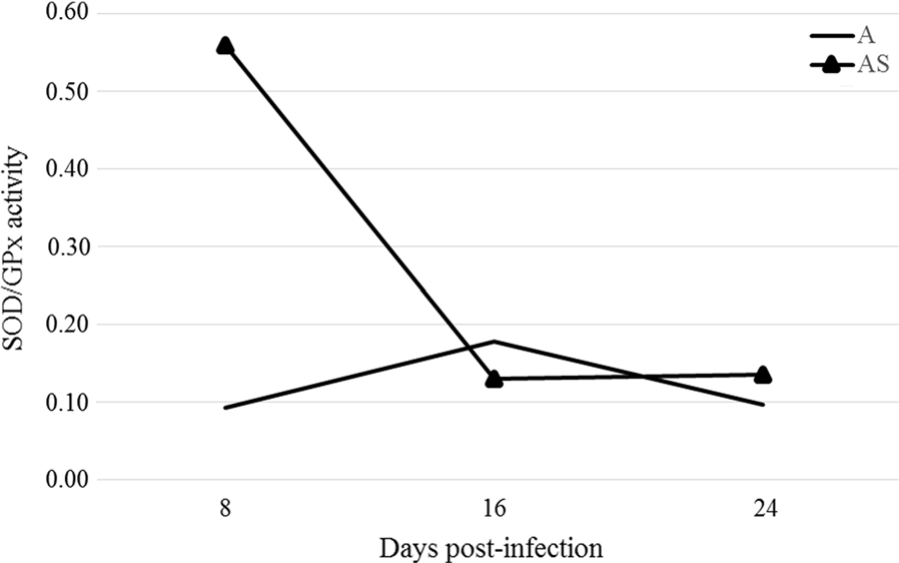
Ratio of activity of superoxide dismutase to glutathione peroxidase in eye samples of immunocompetent and immunosuppressed mice on the 8, 16 and 24 days post-*Acanthamoeba* spp. infection. The graph shows arithmetic means. *Abbreviations*: A, immunocompetent mice; AS, immunosuppressed mice; dpi, days post-infection; GPx, glutathione peroxidase; SOD, superoxide dismutase activity

Eye samples of the immunocompetent mice showed a decrease in SOD/GPx ratio at day 8 post-*Acanthamoeba* spp. infection, then an increase at 16 dpi and a decrease again at 24 dpi (Fig. 3). There were no statistically significant differences in the SOD/GPx ratio in the eye samples collected from mice at days 8, 16 and 24 post-*Acanthamoeba* spp. infection compared to the control group. The immunosuppressed mice showed a statistically significant increase in SOD/GPx ratio at 8 dpi compared to the control group (*U*_(18)_ = 7, *Z* = 2.84*, P* = 0.002), followed by a decrease at 16 dpi (Fig. 3). The SOD/GPx ratio in the immunosuppressed mice eye samples at 16 and 24 days post-*Acanthamoeba* spp. infection was similar; however, at 8 dpi the ratio was more than 6 times higher than in the immunocompetent mice (*U*_(18)_ = 1, *Z* = − 2.30, *P* = 0.02).

## Discussion

Experimental *Acanthamoeba* keratitis may be induced either by direct application of *Acanthamoeba* spp. onto a damaged cornea or by corneal injection. Analyses of histological preparations in *Acanthamoeba* keratitis report corneal oedema, presence of inflammatory cells in the corneal stroma, trophozoites and cysts of *Acanthamoeba* spp. in all epithelial and stromal layers of the cornea, as well as regional stromal necrosis. In addition, the infection may be accompanied by a local necrosis of stromal fibers [24, 25]. In a patient with *Acanthamoeba* keratitis, Kato et al. [26] observed polymorphonuclear leukocytes in the corneal stroma, an abscess in the granulomatous tissue of sclera near the ciliary body, and macrophages and lymphocytes surrounding blood vessels. There was no inflammation of the retina and vascular system [26]. In our study, despite the fact that *Acanthamoeba* spp. was administered intranasally, we found morphological changes in the eyeballs of the immunosuppressed mice, including invagination of the Bowmanʼs membrane into the substantia propria and an increase in the number of layers of stratified non-keratinized squamous epithelium. In the retina, we observed an increased thickness of the outer nuclear layer and an increase in vacuolization inside the outer plexiform layer.

Histological changes in the eyes of mice with disseminated acanthamoebiasis may be a result of changes in the activity of antioxidant enzymes and/or the level of non-enzymatic antioxidants in response to ROS. Saenz de Viteri et al. [27] also found some morphological alteration in the layers of the retina of rabbits due to oxidative stress. These authors observed changes in the thickness of the outer nuclear layer, and an increase in vacuolization inside the outer segments of the photoreceptors. Antioxidant enzymes were also found in the cornea of the eye, lens, iris and ciliary body [18].

The earliest emerging antioxidant enzyme responsible for the removal of peroxide anions is superoxide dismutase. This metalloprotein is found in the eyes in the form of three isoforms. Cu-SOD and Zn-SOD isoforms are present in the cytosol and extracellular fluid and Mn-SOD in the mitochondrion [28]. In the present study, the decreasing activity of SOD over the duration of *Acanthamoeba* spp. infection was observed; however, the differences were not statistically significant. Similar relationships were observed in the lungs of mice infected with the same *Acanthamoeba* strain (AM22). Łanocha-Arendarczyk et al. [5] found a statistically lower SOD activity in immunosuppressed animals at the beginning of the infection (8 dpi). A similar tendency was observed in the lungs of immunocompetent mice 8 days post-infection with *Acanthamoeba* spp., but the differences were not statistically significant. Decreased SOD activity was also found in the plasma of patients infected with *Plasmodium vivax*, and was associated with the intensity of the infection [29]. Raza et al. [30] reported a negative correlation between SOD activity and parasitic activity in patients with subtropical cerebral malaria. Finally, it was found that decreased activity of SOD reduces host responses to oxidative stress caused by inflammatory processes [31].

Catalase is an enzyme that accelerates the decomposition of hydrogen peroxide to water and oxygen. In our study, a significant decrease of CAT activity at 8 and 16 dpi was observed in the eye samples of immunocompetent mice infected with *Acanthamoeba* spp., followed by a rapid increase at 24 dpi. Moreover, CAT activity in the immunocompetent mice at 16 dpi was significantly lower than in the control group. Oxidative stress in the eyes causes a decrease in the activity of antioxidant enzymes, initially CAT and then GPx and SOD, thus increasing the amount of H_2_O_2_ that can damage the cornea of the eye [32, 33]. This was confirmed in our study, where mice with disseminated acanthamoebiasis showed morphological changes in the cornea. Decreased CAT activity was also found in skin leishmaniasis and toxoplasmosis [34]. Finally, Łanocha-Arendarczyk et al. [5] observed an increase in CAT activity in the lungs of immunocompetent mice at 16 days post-*Acanthamoeba* spp. infection.

Glutathione peroxidase is a selenoprotein which has the ability to reduce inorganic and organic peroxides by reducing glutathione, resulting in the formation of an oxidized form of glutathione and water and alcohols, respectively [28]. In our study, in the disseminated acanthamoebiasis with eye infection, GPx activity in the group of immunocompetent and immunosuppressed infected mice was lower than in the control group. A reduction in glutathione peroxidase activity affects the functioning of the antioxidant system. The decrease in activity of these enzymes may be caused by the reaction between the lipid peroxidation products and the selenocysteine residue in the active centre [35, 36]. A decrease in GPx activity was also observed in patients infected with *Plasmodium* spp. [37] and in the lungs of mice infected with *Acanthamoeba* spp. [5].

The oxidized form of glutathione, which is formed as a result of the reaction catalyzed by GPx, may cause the oxidation of thiol protein groups, which leads to their inactivation. Therefore, glutathione reductase found in the mitochondria and cytosol of most cells interacts with GPx. GR catalyzes the reaction to form reduced glutathione [38]. In our study, the eye samples of the immunocompetent and immunosuppressed mice infected with *Acanthamoeba* amoebae showed lower GR activity. Importantly, a decrease in GR activity makes it impossible to reduce the oxidized form of glutathione, which contributes to a decrease in GSH concentration [39]. Glutathione participates in the antioxidative process by detoxifying hydrogen peroxide and organic peroxides. Its presence is crucial for the oxidative balance [40]. In the present study, we observed a decrease and then an increase in GSH concentration in the eye samples of the infected immunocompetent mice. However, in the immunosuppressed mice we found a decrease, an increase, and then again, a decrease in GSH concentration in the eye samples of hosts over the course of the infection with *Acanthamoeba* spp. In a study by Murata et al. [41], increased GSH concentration in host tissues correlates with immunological changes that favor killing parasites, i.e. increased ability to produce IL-12, which differentiates T lymphocytes towards Th1 lymphocytes. Łanocha-Arendarczyk et al. [42] found Th1 induction in *Acanthamoeba* spp. in the immunocompetent and immunosuppressive hosts. Increasing GSH levels in tissues by administration of N-acetylcysteine has been used to protect tissues from *Leishmania major* [43] and *Trypanosoma cruzi* [44].

The antioxidant mechanisms in the eyes of mice infected with *Acanthamoeba* spp. may also depend on the immune status of the host. In the present study, a significantly higher activity of SOD and CAT and GSH concentrations was observed in the immunosuppressed mice infected with *Acanthamoeba* spp. in comparison to immunocompetent infected mice. Methylprednisolone has a strong anti-inflammatory and immunosuppressive effect to inhibit the production of ROS and activate antioxidant mechanisms in various tissues [45]. Increasing the activity of antioxidant enzymes after administration of MPS may protect tissues from the harmful effects of ROS [46]. In the present study, MPS increased the activity of antioxidant enzymes and the non-enzymatic antioxidant concentration in the eye samples of mice infected with *Acanthamoeba* spp. However, in the lungs of mice infected with the same strain of amoebae, no influence of MPS on oxidative stress parameters was reported [5]. AM22 amoebae are pneumotropic, which may cause differences in oxidative stress indices and antioxidative mechanisms between the lung and eye samples.

**Fig. 4:**
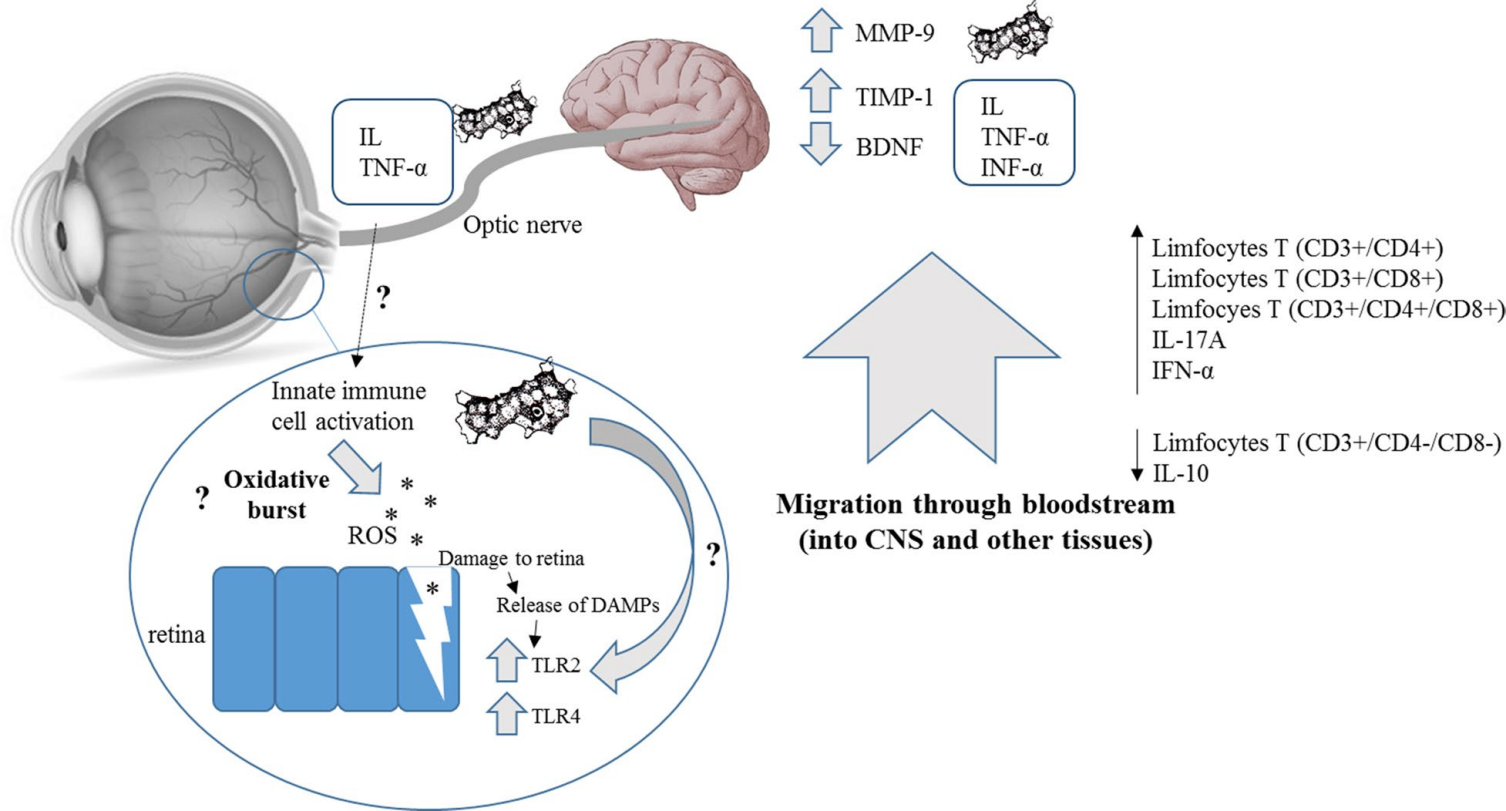
Diagram of possible pathways of pathomechanism of disseminated acanthamoebiasis with eye infection [14, 42, 47, 50, 51] *Abbreviations*: BDNF, brain-derived neurotrophic factor; CD, cluster of differentiation; CNS; central nervous system; DAMPs, damage-associated molecular patterns; IL, interleukin; INF-α, interferon alpha; MMP-9, matrix metalloproteinases 9; NT-4, neurotrophin 4; ROS, reactive oxygen species; TIMP-1; tissue inhibitor of metalloproteinases 1; TLR, Toll like receptors; TNF-α, tumor necrosis factor alpha

The pathomechanism of disseminated acanthamebiasis accompanied by an infection of amebae to the eyeball, is still not fully understood. Łanocha-Arendarczyk et al. [47] in an experimental model of acanthamebiasis observed elevated levels of matrix metalloproteinases 9 (MMP9) and an increased ratio of MMP9 to its tissue inhibitor (TIMP1) in the cortex. Activation of metalloproteinases leads to breaching of the blood-brain barrier and penetration of the morphotic elements of blood into the nerve tissue. Characteristic granulomatous changes in the central nervous system result from the accumulation of CD4+ and CD8+ T lymphocytes, B lymphocytes, plasma cells, macrophages and amebae [48]. This presence of immune system cells in the brain suggests the participation of IFNγ, IL-1α, IL-6 and TNF-α [49]. Łanocha-Arendarczyk et al. [50, 51] also observed a decrease in the activity of brain-derived neurotrophic factor (BDNF) and neurotrophin 4 (NT4) in the cortex of *Acanthamoeba* spp.-infected mice, which correlated with the duration of infection. Elevated MMP levels and decreased neurotrophin levels may contribute to remodeling of the extracellular matrix (ECM), which may allow cell migration, including that of *Acanthamoeba* spp. Previous research [14] and this present study indicate that amebae are able to migrate through the optic nerve from the brain to the eyes. A pro- and antioxidant imbalance in the mouse eyeball may result from the presence of amebae in retinal structures, as well as from the activation of other immune cells. We speculate that histopathological changes in the retina induced by increased ROS may contribute to a release of damage-associated molecular patterns (DAMPs) and activation of Toll-like receptors, as shown by changes in the expression of TLR2 and TLR4 in mice eyeballs with disseminated acanthamoebiasis [14]. However, TLRs can also be activated directly by *Acanthamoeba* spp. (Fig. 4).

## Conclusions

The results of the study showed that the activity of enzymatic antioxidants and the content of non-enzymatic antioxidants in the host organism change over the course of disseminated acanthamebiasis. In the immunocompetent mice, these changes manifested themselves in the decreased activity of catalase and glutathione reductase and increased concentration of glutathione. Immunosuppressed hosts showed decreased activity of glutathione peroxidase and increased glutathione concentration. We speculate that pro-oxidant/antioxidant imbalance was probably caused by the activation of innate immune cells in response to infection with *Acanthamoeba* spp. However, the pathogenesis of disseminated acanthamoebiasis with eye infection is still poorly understood and documented. In further studies, we suggest examining the parameters of oxidative stress and antioxidant enzymes in different ophthalmic tissues. Moreover, it is necessary to analyse the immunophenotype of cells in the eyes to confirm or reject the theory that *Acanthamoeba* spp. activates the innate immune cells, which lead to oxidative burst.

## Data Availability

The datasets used and analysed during the present study are available from the corresponding author upon reasonable request.

## Abbreviations

A: immunocompetent *Acanthamoeba* spp.-infected mice
AK: *Acanthamoeba* keratitis
AM 22: amoebic strain no. 22
AM: arithmetic mean
AML: acute myeloid leukaemia
AS: immunosuppressed *Acanthamoeba* spp.-infected mice
BDNF: brain-derived neurotrophic factor
C: immunocompetent uninfected control group mice
CAT: catalase
CD: cluster of differentiation
CS: immunosuppressed uninfected control group mice
CNS: central nervous system
DAMPs: damage-associated molecular patterns
dpi: days post-*Acanthamoeba* spp. infection
EDTA: ethylenediaminetetraacetic acid
ECM: extracellular matrix
GAE: granulomatous amebic encephalitis
GPx: glutathione peroxidase
GR: glutathione reductase
GSH: glutathione
GSSG: oxidized glutathione
IL-12: interleukin 12
MBP: mannose binding protein
MMP: matrix metalloproteinases
MPS: methylprednisolone sodium succinate
NADP: nicotiamide adenine dinucleotide phosphate
NADPH: reduced nicotiamide adenine dinucleotide phosphate
NN: non-nutrient agar
NT-4: neurotrophin 4
*P*: level of significance
PBS: phosphate-buffered saline
ROS: reactive oxygen species
SD: standard deviation
SOD: superoxide dismutase
TIMP-1: tissue inhibitor of metalloproteinases 1
Th1: type 1 T helper
TLR2: toll-like receptor 2
TLR4: toll-like receptor 4
TNF-α: tumor necrosis factor alpha
